# Perceptions and attitudes of black men in a rural district of South Africa towards depression and its treatment

**DOI:** 10.4102/safp.v64i1.5557

**Published:** 2022-07-28

**Authors:** Hlabje C. Masemola, Saiendhra V. Moodley, Joyce Shirinde

**Affiliations:** 1 School of Health Systems and Public Health, Faculty of Health Sciences, University of Pretoria, Pretoria, South Africa

**Keywords:** depression, perceptions, attitudes, mental health, help-seeking, treatment, South Africa

## Abstract

**Background:**

Depression is a major contributor to the overall global burden of disease, impacting social life, family life and occupational functioning if left untreated. Despite its high prevalence and morbidity, the evidence suggests that men are hesitant to seek help, with a large percentage remaining undiagnosed. This study aimed to determine the attitudes and perceptions related to depression and its treatment amongst black men in a rural district of South Africa.

**Methods:**

The design used was an exploratory descriptive qualitative design. Participants were selected by purposive sampling. Individual semistructured interviews were conducted, which were audio-recorded and transcribed verbatim. The data were subsequently analysed thematically to develop themes and subthemes.

**Results:**

Participants described depression as a psychological problem associated with lack of sleep, loneliness, feeling unwanted, increased stress, deep sadness, weight loss, forgetfulness, crying over small things and lack of concentration. Collectively, the interviews with participants showed a good understanding of the psychosocial determinants of depression but exposed a lack of awareness of its biological determinants. A large proportion (*n* = 13; 68.4%) of participants reported not having knowledge of available services in their area for people seeking treatment for depression. Barriers to help-seeking behaviours were fear of social stigma, fear of expressing their feelings, gender norms and stereotypes and lack of trust in others.

**Conclusion:**

Interventions such as support groups and mental health awareness programmes to counteract personal perceptions may help to improve and expand the effectiveness of depression treatment. The results highlight the future need to raise awareness of depressive symptoms and expand health outreach programmes.

## Introduction

Mental illnesses are recognised as a public health concern in both developed and developing countries.^1^ The significance of mental and behavioural disorders is highlighted by these disorders being amongst the most significant causes of morbidity in primary care and the substantial disability they produce.^2^ Depression is a significant contributor to the overall global burden of disease, impacting social life, family life and occupational functioning if left untreated.^3^ Depressive disorders were ranked as one of the three leading causes of the global burden of disease in 2017 and it is expected to rise and become the leading cause by 2030 affecting low-, middle- and high-income countries.^4^ Globally, depressive disorders are ranked as the single largest contributor to nonfatal health loss, with 7.5% of all years lived with disability (YLD).^4^ The 2019 Global Burden of Disease Study ranked depressive disorders as the sixth leading cause of YLD globally in the 25–49 years age group.^5^ Depression also contributes substantially to suicide deaths, with suicide deaths globally close to 800 000 per year.^4^

Depression is highly prevalent, with more than 322 million people affected irrespective of gender.^4^ An increase of 18.4% in people living with depression has been estimated between 2005 and 2015.^4^ The proportion of the world’s population with depression was estimated to be 4.4% in 2015.^4^ According to the World Health Organization (WHO), the prevalence in South Africa was estimated to be 4.6% of the population in 2015.^4^ South Africa is characterised by multiple epidemics of depression, with heterogenic distribution within the country that is possibly driven by several epidemiological and socio-economic cofactors.^6^ Research findings highlight spatially localised micro-epidemics rather than a national epidemic of depression in South Africa.^6,7^ Based on a 2012 South African national population survey, 27.1% of South Africans were reported to have depressive symptoms, whilst a 2015–2016 South African national population survey indicated this was 13.0%.^6,8^ In a primary health care clinic-based study of HIV-infected individuals in Johannesburg, South Africa, the prevalence of depressive symptoms was high, with 30.0% of individuals experiencing probable depression.^9^

Attitudes and beliefs about depression are influenced by what individuals understand about mental illnesses, interacting with those affected by mental illness, cultural stereotypes, information received about mental illnesses and familiarity with practices and restrictions associated with mental illness.^10^ People’s understanding and interpretation of mental health and illness differ from culture to culture, and their help-seeking behaviour (or lack thereof) is explained by their perceptions of illness.^11^ In response to the WHO Comprehensive Action Plan 2013–2020, which aims to promote mental well-being and prevent mental disorders such as depression,^12^ the South African National Mental Health Policy Framework and Strategic Plan 2013–2020 was developed in acknowledging a lack of public awareness of mental health and illness, as well as widespread stigma against those with mental illnesses such as depression.^13^

Men generally seek mental health treatment less often than women.^14^ This may explain why there are higher rates of suicide in men.^15^ Globally, black males are less likely to seek help than other races.^16^ Comparing Caucasians and African-Americans, African-Americans are considered less likely to seek help for depression and have less access to culturally compatible and competent providers, with stigma as a prominent barrier to help-seeking.^10,17^ It has been suggested that men position themselves based on hegemonic masculinity (conceptualised as the superior practices in society relating to the conduct of boys and men^18^) as a pervasive aspect of their actions, and for them to be accepted as a man in society, they must exhibit fearlessness, emotional endurance and toughness as well as engage in risky behaviours which affect help-seeking behaviour.^18,19^ Furthermore, Mthethwa^19^ indicated poor access and knowledge of psychological services within the general black population, especially amongst males. Taking the disease burden of depression and the multifaceted nature of the problem into account, this study aimed to explore the attitudes and perceptions of black men in Waterberg District, South Africa, towards depression, health service attendance for depression, and its treatment.

## Research methods and design

### Study design

To understand human thoughts and actions as well as to obtain deep insights into the perception and attitudes of black men, a qualitative approach driven by the naturalist interpretive paradigm was used.^20^ This paradigm allowed for the examine life experiences to understand the phenomenon and give meaning to it.^21^ In this study, an exploratory descriptive design was used to explore and describe the perceptions and attitudes of black men towards depression and its treatment.^22^ Individuals came up with the subjective meanings of their experience, which led to the researcher looking into the complexity of views rather than a few categories of ideas.^23^ Qualitative research provides comprehensive data about thoughts, feelings and/or human observations to establish meaning from life experiences, which made it suitable for this study.^24^ There was diversity in the research team in terms of gender and race representation. The researchers recognised that their background and work experience in the health fraternity influence their interpretation of the data, and they positioned themselves in the research to take into account how their understanding results from their own personal, historical and cultural experiences and to ensure data were not compromised and participants were not put at risk.^23^

### Study setting

Participants were recruited in Lephalale in the Waterberg District, Limpopo province, South Africa. The Waterberg District Municipality is classified as 90% rural.^25^ The study site lies close to the Botswana border, surrounded by mining, electricity production, tourism, agriculture and game farming sectors. A private-sector medical centre was used to conduct the interviews because of its proximity to the minibus taxi rank and easy access to many people living in the area. Interviews were conducted in a private environment in the centre.

### Study population and sampling strategy

The study population was black men living in the Lephalale area. Inclusion criteria for this study required the interviewed participants to be black, male, aged between 18 and 59 years and living in the area for at least 5 years. The target was to include a minimum of 15 participants in the study and to continue to recruit participants if data saturation had not been reached at that point. Data saturation was eventually reached at 19 participants. A purposive sampling strategy was used. This is a nonprobability sampling method where the researcher selects participants according to their judgment about who will be most informative.^26^ The participants recruited were individuals who were either visiting or working at a shopping centre or those visiting the medical centre for consultations.

### Data collection

The researcher conducted face-to-face semistructured in-depth interviews with the recruited individuals that intended to explore their views and opinions. The interviews were led by an interview guide. The interviews were audio-recorded and later transcribed in English by a language practitioner. Study participants signed a consent form to participate in the study and were scheduled in advance at a designated time at the medical centre, and the interviews ranged from approximately 10 to 25 min, with the majority lasting for approximately 15 min. Member checking was offered to all participants by giving them the transcripts to make corrections. When contacted, none of the participants felt that it was required to review the transcripts and were all satisfied with their responses to the initial interview. The researcher also used rich data and prolonged engagement to ensure trustworthiness. To ensure the flow of the interviews was not disrupted, the sessions were conducted in a setting that enabled safety, privacy and confidentiality. There was no external noise from other people or devices, such as mobile or office phones that could interrupt sessions. Given the coronavirus disease 2019 (COVID-2019) pandemic, all participants were hand sanitised with an alcohol-based sanitiser before and after the interview, face masks were worn for the period of the interview and a social distance of at least one metre was maintained between each participant and the researcher as infection prevention and control measures.

### Data analysis

In facilitating analysis, the transcripts were checked for accuracy. Thematic analysis was used to make sense of the qualitative data, and the researcher was immersed in the data in searching for meanings and essential patterns in the analysis of insightful findings.^27^ To ensure interpretive epistemology during the process of reading and coding the transcripts, the researcher made a conscious effort to set aside pre-existing expectations and ideas and to place themselves in the position of the participants.^28^ ATLAS.ti 9 software was used in the coding of text data to present several themes until no new themes could be generated. The interview transcripts were read and coded by the researcher.

### Ethical considerations

The Faculty of Health Sciences Research Ethics Committee of the University of Pretoria approved the study proposal (reference number 98/2021). A formal letter of permission to conduct the study was obtained from Lethabong Medical Centre. We obtained informed consent from participants after explaining the purpose of the study and informed them that the study was voluntary, and confidentiality was maintained by omitting their personal information in the transcripts.

## Results

A total of 19 black men aged between 18 and 59 years were interviewed between 17 June 2021 and 15 July 2021. The mean age of the sample was 34 years (s.d.: 8.46), with a median age of 33 years. Most men had completed a college diploma (*n* = 10; 52.6%) or had some school education (*n* = 7; 36.8%). Employment status in the sample was currently employed full-time (*n* = 12; 63.2%) or part-time (*n* = 2; 10.5%), and the others were unemployed (*n* = 5; 26.0%). The majority of participants indicated that they had never been married (*n* = 10; 52.6%). Table 1 displays the full sociodemographic characteristics of the study sample.

**TABLE 1:** Sociodemographic characteristics of the participants (*N* = 19).

Variable	*n*	Percentage (%)
**Marital status**		
Married	6	31.6
Living with partner	2	10.5
Separated	1	5.3
Never been married	10	52.6
**Education**		
Primary school	1	5.3
High school	7	36.8
College diploma	10	52.6
University degree	1	5.3
**Current work status**		
Full-time employment	12	63.2
Part-time employment	2	10.5
Unemployment	5	26.3

The results of the interview data revealed four themes based on the thematic analysis. The four themes were identified from the participants’ responses: (1) perceptions related to the symptoms and causes of depression, (2) perceptions related to the treatment of depression, (3) barriers to help-seeking behaviours and (4) practices facilitating help-seeking behaviours. Perceptions related to causes of depression as a theme consisted of subthemes that are shown in Table 2.

**TABLE 2:** Emerging themes and subthemes.

Theme	Subtheme
Perceptions related to symptoms and causes of depression	Stress in relationships Financial problems Lack of family support Unemployment
Perceptions related to the treatment of depression	-
Barriers to help-seeking behaviours	-
Practices facilitating help-seeking behaviours	-

### Perceptions related to symptoms and causes of depression

When participants were asked what the word depression meant and what were its symptoms, the most frequent responses reported depression as a psychological problem associated with lack of sleep, loneliness, feeling unwanted, stressing a lot, deep sadness, loss of weight, forgetfulness, crying over small things and lack of concentration. The common responses related to perceptions of depression charted into known depressive symptoms, characterised by depressed mood, loss of interest and enjoyment, poor self-esteem and self-confidence, feelings of guilt and unworthiness, disturbed sleep or appetite and poor concentration.

The causes of depression were perceived by participants as being mainly social and financial problems. The causes identified as key were stress in relationships, financial problems, lack of family support and unemployment. All participants highlighted the external causes of depression amongst black men, and this raised alarm of possible events that build on and influence each other towards depression in men. Collectively, the interviews with participants exposed a limited understanding of the causes of depression. The causes of depression were associated with psychosocial causes, and there was no mention of biological causes.

#### Stress in relationships

Stress in relationships as a cause of depression generated more consensus (*n* = 14; 73.5%) amongst the participants, and it was mentioned that stress makes it difficult to maintain their relationships. We noted through the interviews that most participants understood the impact of stress on their relationships. In this context, participants specifically referred to their relationships with their partners, siblings and close relatives. The analysis showed that the stress comes from arguments, misunderstandings and feelings of neglect between couples. The following quotes illustrate stress in relationships:

‘[… *L*]et’s say, in terms of families, a man is abused by his wife and he doesn’t tell anyone about it.’ (Participant 7, 43 years old, 6 July 2021)

‘[… *W*]hen people that you spend your life or live with mistreating you.’ (Participant 18, 41 years old, 15 July 2021)

‘Or maybe a guy who is in a relationship or a marriage, and you find that his partner or wife is mistreating him or even leave him …’ (Participant 6, 24 years old, 3 July 2021)

‘Another thing is that if you happen to have arguments with your spouse and you can’t solve your problems, you might end up being depressed.’ (Participant 9, 36 years old, 8 July 2021)

‘[… *S*]ocial problems, whereby there are misunderstandings between you and your partner.’ (Participant 11, 40 years old, 8 July 2021)

‘Having too many expectations on many things such as relationships.’ (Participant 13, 38 years old, 9 July 2021)

‘Relationships can be one cause.’ (Participant 14, 28 years old, 9 July 2021)

‘It is mostly caused by relationships, whereby you find that you don’t get along with your partner.’ (Participant 16, 22 years old, 13 July 2021)

#### Financial problems

Nine of the 19 (47.4%) participants mentioned that financial problems were the main contributing factors to depression, which made it difficult for them to pay for their expenses and forced them to survive with the little they had. The following quotes demonstrate financial problems:

‘Ahh, number one is financial problems, whoo!’ (Participant 5, 25 years old, 22 June 2021)

‘Normally, the ones that we are experiencing are financial problems. I think financial problems play a very big role in depression.’ (Participant 11, 40 years old, 8 July 2021)

One participant **emphasised** an interesting explanation for the kind of financial problems and the implications thereof, stating the following:

‘And then some because we are incapable of controlling our budget and our money. If the money is not enough, you might be depressed because you can no longer afford some of the things. The lifestyle changed drastically.’ (Participant 18, 41 years old, 15 July 2021)

#### Lack of family support

Six of the 19 (31.6%) participants mentioned lack of family support as one of the causes of depression. There was also an indication of dissatisfaction in how parents treat male children from childhood. The following excerpts elaborate on the negative behaviour that men may face in their families:

‘Some people do not get any kind of support from their families when they are growing up, and that can affect them mentally later in life.’ (Participant 2, 38 years old, 22 June 2021)

‘Being the head of the family means that you are not even supposed to cry anymore; you are supposed to be strong all the time, which is not humanly possible.’ (Participant 4, 22 years old, 22 June 2021)

‘Let’s say you are young, and your mom or parents die, and you don’t receive any kind of support or guidance from your relatives.’ (Participant 8, 39 years old, 8 July 2021)

#### Unemployment

Five of the 19 (26.3%) participants were concerned with the high level of unemployment, which is believed to be a contributing factor to depression because it makes it difficult to look after other family members, contributing to feeling useless. The quotes below elaborate on the responses related to unemployment:

‘If you are unemployed and you have kids to take care of, that could cause depression.’ (Participant 9, 36 years old, 8 July 2021)

‘For example, if you are a family man and you are unemployed, unable to take care of your family, you would feel useless.’ (Participant 13, 38 years old, 9 July 2022)

### Perceptions related to the treatment of depression

Overall, participants revealed good knowledge of how health service providers can assist people who are depressed. Psychologists and social workers were identified as the main health service providers that people could consult for personal problems that could lead to depression. Nine of the 19 (47.7%) participants were open to talking to a professional about their problems. The following quotes highlight the perceptions related to the treatment of depression:

‘There are psychologists at the Ellisras Hospital. That’s one place that I know has psychologists. I think Witpoort also has psychologists who can help people with depression.’ (Participant 7, 43 years old, 6 July 2021)

‘You don’t have to walk very long distances to get help, even if you are in rural areas. Social workers and psychologists are available.’ (Participant 8, 39 years old, 8 July 2021)

Alarmingly, 13 of the 19 (68.4%) participants reported not having an understanding of available services for people who are depressed in their local area. Although participants were aware that health professionals help depressed individuals, they responded that they never heard of those services in their areas, never bothered to find out or simply did not know where to receive information:

‘I don’t know of any. Maybe they are there and I just don’t know about them. I have never been close with anyone who is experiencing this problem. I don’t know.’ (Participant 17, 42 years old, 14 July 2021)

Nine of the 19 participants (47.4%) indicated that counselling is the most common treatment option that could work for people who are depressed. Participants indicated that most people who undergo counselling and have a good understanding of their depressive symptoms have enhanced prospects for recovery:

‘For treatment, the first people you should consult are psychologists. They would be able to give you counselling, and where there is a need, they will refer you to doctors who specialise in mental health.’ (Participant 2, 38 years old, 22 June 2021)

‘[… *T*]hey come back healthier because they offer them counselling.’ (Participant 18, 41 years old, 15 July 2021)

‘I will accept it positively because I want to be safe [healed]. I will accept it with immediate effect because it will save my life.’ (Participant 2, 38 years old, 22 June 2021)

‘I would be open to talking to a professional if I know that a professional specialises with problems that are similar to mine.’ (Participant 8, 39 years old, 8 July 2021)

Although the majority of participants (*n* = 16; 84.2%) were willing to access professional help should they need it, only 6 of the 19 (31.6%) mentioned antidepressants as a treatment option and only 2 out of those 6 indicated that antidepressants are effective in treating depression; the remaining 4 still questioned pharmacological management. A large proportion (*n* = 13; 68.4%) of participants showed no understanding of the treatment options available:

‘I was once referred to an industrial psychologist, but I didn’t find any use of it because I felt that the same questions they are asking me are the same questions I am asking myself.’ (Participant 14, 28 years old, 9 July 2021)

‘I am not against pills and drugs, [*and*] they have their benefiting factors; however, a mental state is a mental issue; you cannot out-drug what people think.’ (Participant 5, 25 years old, 22 June 2021)

‘I am not a medical doctor, but I don’t think that there is any treatment that can work for depression. Depression is something that has got to do with the mind. You cannot treat the mind unless if you say maybe … unless if depression was a disease inside …’ (Participant 12, 33 years old, 9 July 2021)

‘I think they are there. The treatment, though, would be to talk, not medication. I think that’s what can help.’ (Participant 19, 24 years old, 15 July 2021)

‘I think therapy, though I don’t believe in it. Therapy and medication. But the problem is the dosage of medication doctors gives … I don’t want that depressed person to depend on the medication, and then when they are not taking all the medication now they just go back to…’ (Participant 1, 31 years old, 17 June 2021)

It was also found that most of those interviewed (*n* = 16; 84.2%) were accepting of men who are depressed and were willing to offer support and compassion to men who are depressed. The responses were attributed to the principle of *ubuntu*, a South African term recognising respect, communality and humanness:

‘I think I would want to be close to him – he is a human being, after all.’ (Participant 2, 38 years old, 22 June 2021)

‘I would be; I won’t abandon him. I would be close to him and try to advise him on what he can do regarding his problems.’ (Participant 7, 43 years old, 6 July 2021)

‘I would be friends with them because the more we know what other people go through in life, the better we become, and we would know what to do should we go through the same.’ (Participant 13, 38 years old, 9 July 2021)

‘I can’t say they are weak. I can feel sorry for them because this is something that they came across; they were not born with it.’ (Participant 19, 24 years old, 15 July 2021)

‘I have this belief that we have to always ask each other how we are doing, because that’s where we will be able to know if someone is okay or not.’ (Participant 13, 38 years old, 9 July 2021)

### Barriers to help-seeking behaviours

Barriers to help-seeking behaviours were fear of being seen as weak by the society (social stigma), fear of expressing their feelings, gender norms and stereotypes and lack of trust in others. Based on the responses, most men have fears of being seen as weak by society. Participants expressed the belief that society has put so much pressure on men that it makes them wary of what other people will say if they consider seeking help. Most participants mentioned that as men are the head of the household, they are expected to be tough as the entire family looks up to them. The following quotes reflect their experiences:

‘I think society has put a lot of burden upon men.’ (Participant 1, 31 years old, 17 June 2021)

‘We think we should be strong, and people also expect us to be strong.’ (Participant 13, 38 years old, 9 July 2021)

‘Society also – what are people going to say about me?’ (Participant 14, 28 years old, 9 July 2021)

Expressing feelings towards others is believed to be a sign of weakness or not having everything under control. Participants pointed out that men are shy in expressing their feelings and they hold a deep view that they will be judged. There is also an element of pride that comes with not being willing to share personal problems with others. It is believed that men do not trust easily and always question if the information that they share with others will remain confidential or not:

‘They don’t want to tell other people their problems because they think their problem will not be safe.’ (Participant 3, 28 years old, 22 June 2021)

‘They are worried about what people will say if they tell them their problem.’ (Participant 19, 24 years old, 15 July 2021)

‘[… *W*]e also are shy to open up and also we are worried that I am going to tell Dr [*name*] … about my problems, and then he will be driving here seeing me. … It looks like he told everyone about my problems.’ (Participant 12, 33 years old, 9 July 2021)

Some participants said that as men, they are expected not to cry and they need ‘to toughen up’ to validate themselves as men. They mentioned that it is expected of them to behave differently from women, and it is something that comes from upbringing:

‘We are from an upbringing where they tell you, “you need to be like this; you do this; you don’t do that; this is for women; this is for men.”’ (Participant 1, 31 years old, 17 June 2021)

‘Whenever you get to a point where something is too hard for you, when you are young, you will express and you say, “this thing I didn’t like,” and whatever, and the first thing you will do, whether it’s your mother, whether it’s your father or your uncle, they say, “no, you must toughen up.”’ (Participant 5, 25 years old, 22 June 2022)

‘In Sepedi culture, they say a man must not be like a woman and cry out his problems.’ (Participant 8, 39 years old, 8 July 2021)

‘I have to prove that I am a man; I can go through this, if whoever went through this … but forgetting that I am not the other person.’ (Participant 14, 28 years old, 9 July 2021)

Being treated by a woman or younger professional was also a barrier to seeking help. Participants cited concerns about feeling uncomfortable discussing personal problems with women:

‘When they go consult, they would want to be helped by a man and not a woman. If they find a woman, they might not be comfortable or even leave without getting help.’ (Participant 3, 28 years old, 22 June 2021)

‘Another thing is that you may find that a psychologist is a young person, and as an older person I would be reluctant to go to them; they are young and I am older.’ (Participant 7, 43 years old, 6 July 2021)

Three participants mentioned the cost of treatment as impeding help-seeking. They alluded to treatment for depression as it can sometimes involve out-of-pocket expenditure, even for those with private medical insurance (medical aid), especially when these funds are depleted:

‘Unless if maybe the barrier can be financial reasons, or maybe my medical aid is exhausted.’ (Participant 12, 33 years old, 9 July 2021)

‘If it’s costly, then it’s going to be a problem.’ (Participant 14, 28 years old, 9 July 2021)

‘The only obstacle would be finances.’ (Participant 15, 26 years old, 9 July 2021)

One participant firmly believed that depression is deeply rooted in cultural beliefs and often when something unusual occurs, they think of witchcraft and often seek help from a *sangoma* (traditional healer).

‘The cultural beliefs. Some like … in our culture, if some things are happening, the first thing that we think of is witchcraft. We don’t think out of the box, especially because such kinds of things we normally used to say that the disease is for white people. In our culture, it will be like, “it’s witchcraft, then you must go to a *sangoma.*”’ (Participant 11, 40 years old, 8 July 2021)

### Practices facilitating help-seeking behaviours

Many participants mentioned that being open and willing to talk about their problems will increase their chances of seeking help for depression, as the advice they may receive ultimately enables them to seek help. The following excerpts elaborate on practices facilitating help-seeking behaviours:

‘We need to allow people to open up about their problems.’ (Participant 2, 38 years old, 22 June 2021)

‘We need to learn to talk – that’s the number one solution.’ (Participant 8, 39 years old, 8 July 2021)

Participants showed consensus on the view that support groups were an opportunity to facilitate help-seeking behaviours. Having support groups, health awareness programmes and health literacy were regarded as good initiatives to address the increase in depression amongst men to avoid further complications:

‘We can create men’s support groups where we could share our problems and have someone who is a professional, who can help us to find solutions.’ (Participant 6, 24 years old, 36 July 2021)

‘To prevent depression from spreading, we need to have awareness programs in communities, and advise them to talk whenever they have problems and to also see psychologists and give them information and details about those.’ (Participant 7, 43 years old, 6 July 2021)

## Discussion

This study sought to determine the attitudes and perceptions related to depression amongst black men. Within a rural South African setting, black men in this study report a limited understanding of depression. Participants were only aware of the causes of depression associated with multiple interacting psychosocial factors such as stress in relationships, financial problems, lack of family support and unemployment, and there was no mention of biological causes of depression. These results are inconsistent with those of the study conducted in a mixed-race British sample in Britain on the beliefs about the causes and cures of depression, which showed that factors causing depression were labelled God or fate, environmental, health, self-obsession, brain, genetics and parents.^29^

Our study shows awareness by participants of the health service providers that can help people who are depressed; however, there was poor knowledge of services available for depressed people in their local area. According to Herman et al.,^30^ a considerable amount of South Africans experience symptoms of psychological distress, but there is a substantial treatment gap, with very few receiving the care they need because of the significant lack of access to mental health care services. Psychologists and social workers were identified as the main health professionals that people can consult for personal problems that could lead to depression. The study results are consistent with the findings of the previous research conducted locally, which indicated that knowledge and accessibility of local psychological services were low within the black population generally but particularly amongst males.^19^

Participants in this study showed limited understanding of pharmacological treatment available and the benefits of the treatment options. Contradictory results were obtained in a Canadian study, where high uptake and knowledge of treatment were found in participants who had a better-quality doctor–patient relationship.^31^ It is likely that the difference with the Canadian study is because of the patient–doctor relationship amongst men or men not seeking help. Fears of being seen as weak by society (social stigma), fear of expressing their feelings, gender norms and stereotypes and lack of trust in others appeared to hinder help-seeking behaviours. Our findings reinforce the notion from a systematic review by Seidler et al.^32^ of ‘the problematic impact of conformity to traditional masculine norms on the way men experience and seek help for depression’.

Whilst support groups, health awareness programmes and health literacy were regarded as good initiatives to encourage help-seeking behaviour for depression, barriers regarding the cost of treatment and being treated by a woman or younger professionals were also mentioned. There are also major barriers to depression treatment noted in the literature, including affordability of care, acceptability of treatment, mistrust of mental health providers and norms linked to masculinity.^33^ The results of this study are commonly seen in international studies where men who live with depression and anxiety attend support groups, which are low cost and a good starting point for engaging other men and may work well in combination with one-to-one talking therapies.^34^

It is frequently assumed that men are commonly uninformed about depression being a health issue of concern and are reluctant to seek professional help if they became depressed. The results revealed that the participants interviewed understood the features of depression. The majority cited depression as a lack of sleep, loneliness, feeling unwanted, stressing a lot, deep sadness, loss of weight, forgetfulness, crying over small things and lack of concentration. These descriptions are consistent with symptoms mentioned in the Diagnostic and Statistical Manual of Mental Disorders – Fifth Edition (DSM-V).^35^ Although participants were aware of depression’s symptoms, research suggests that black South Africans also receive treatment from a traditional healer for emotional and mental health concerns despite not having a lifetime DSM-IV diagnosis.^36,37^ Most participants in this sample communicated willingness to seek professional help to assist with their problems, displaying a positive attitude towards help-seeking behaviours. These findings are supported by results from other studies, as it has been observed that Canadian men identify depression as a grave health concern amongst men, contradicting the predominant perception that there is a lack of awareness amongst most men of depression being a major health problem.^38^ In contrast to Canadian men identifying depression as a grave health concern, more negative attitudes were found in black men, where a reasonable amount of self-stigma was a major barrier to seeking help for mental health issues.^39^

Although most participants indicated a willingness to seek professional help for their problems, few participants in our study would have sought treatment for depression despite needing the treatment, citing not having knowledge of health services in their area, social stigma, fear of expressing their feelings, gender norms and stereotypes, affordability of the treatment and lack of trust in health service providers as reasons. The lack of information and knowledge of mental health services offered has been noted in other studies, which may delay help-seeking behaviour in men.^19^ When asked about the acceptability of depression treatment, participants were open to ‘talk therapy’ but either had no knowledge of antidepressant medication or believed the pharmacological treatment would not be effective. These findings are consistent with a study that reported African-American men presenting with similar barriers may resist seeking treatment.^33^

As with a past study, several men in this study also reported social stigma as an obstacle preventing appropriate action in accessing the health system for the necessary care.^40^ The men in the study believed that society would judge them should they know about their potential depression. When men feel vulnerable, it is hypothesised that they fear a loss of status and power, which results in them overconforming to masculine norms.^40^ Men in this study also validated gender-socialised beliefs that whilst women can express their feelings, being emotional is emasculating, a stance supported in the literature.^40^ Masculine social norms such as emotional control and self-reliance make it challenging for men to seek help when they are in need or acknowledge their subjective distress.^41^

Another important finding that emerged from this study is that participants expressed their wish to talk about their problems in nonjudgemental support groups and believed that support groups and having awareness programmes in their communities would prevent depression and ultimately improve their help-seeking behaviours. Despite few studies that examine the effectiveness of support groups, available evidence suggests that peer support interventions help reduce depressive symptoms.^42^

This study has provided valuable insights into the perceptions of depression in a population in which help-seeking may be a potential concern. However, there were some limitations. The participants were aware that they were being interviewed by a health professional and may to some extent have provided socially desirable answers. The study used nonprobability sampling, which makes it difficult to know how well the population is represented. The interviews were conducted in a private health care facility, with some participants recruited whilst visiting the facility, and the results may over-represent private users when compared to the rest of the district. The generalisability of the study findings beyond the study population may not be possible.

## Conclusion

The study indicated a limited understanding of the causes of depression and its treatment. Whilst participants appeared to have a good understanding of the psychosocial determinants of depression, there was a lack of awareness of its biological determinants. Though the findings highlighted a generally positive attitude towards help-seeking for depression, social stigma and gender stereotypes were identified as major barriers which need to be addressed to promote actual help-seeking behaviour. Interventions such as support groups and mental health awareness programmes to counteract personal perceptions may help to improve and expand the effectiveness of depression treatment. The results highlight the future need to raise awareness of mental services in local areas and expand outreach health programmes.
